# Identification of psoriasis-associated hub genes by integrated transcriptomics and machine learning

**DOI:** 10.3389/fimmu.2026.1937515

**Published:** 2026-10-05

**Authors:** Yaqi Zhao, Xiao Liang, Mingwei Tang, Lin Qiao, Wei Bai, Can Huang, Mengtao Li, Xiaofeng Zeng, Xiaomei Leng

**Affiliations:** 1 Department of Rheumatology and Clinical Immunology, Peking Union Medical College Hospital, Chinese Academy of Medical Sciences, Peking Union Medical College, Beijing, China; 2 National Clinical Research Center for Dermatologic and Immunologic Diseases (NCRC-DID), Ministry of Science & Technology, Beijing, China; 3 State Key Laboratory of Complex Severe and Rare Diseases, Peking Union Medical College Hospital, Beijing, China; 4 Key Laboratory of Rheumatology and Clinical Immunology, Ministry of Education, Beijing, China

**Keywords:** bioinformatics analysis, hub gene, machine learning, molecular docking, psoriasis

## Abstract

This study aims to systematically identify key hub genes in psoriasis pathogenesis through the integration of bioinformatics, machine learning and experimental validation methods. Four psoriasis microarray datasets were retrieved from the GEO database, with differentially expressed genes identified through comparative analysis. A dual machine learning approach screened core hub genes, whose expression levels and diagnostic efficacy were validated in an independent cohort. CIBERSORT was employed for immune cell infiltration analysis, whilst a multi-database approach constructed an mRNA-miRNA-lncRNA regulatory network. Molecular docking technology assessed interactions between the environmental pollutant bisphenol A, predicted drugs, and hub genes. Finally, an *in vitro* psoriasis model was established by stimulating HaCaT cells with the M5 cytokine mixture, with hub gene expression validated via RT-qPCR. Results show that 77 co-differentially expressed genes were identified in psoriasis. Machine learning screened five key hub genes: CARHSP1, HPSE, PCLAF, SERPINB4, and SPRR2C. These were significantly upregulated in the validation cohort and demonstrated strong discriminative value for distinguishing psoriatic lesional skin from paired non-lesional skin, rather than serving as standalone diagnostic biomarkers for psoriasis. Immune cell infiltration analysis revealed extensive associations with immune subpopulations including T cells and macrophages. Molecular docking computationally predicted a potential binding interaction between the environmental pollutant BPA and the hub gene HPSE, which awaits future experimental validation. Concurrently, Genistein and HPSE were suggested a potential interaction that awaits functional validation. *In vitro* experiments confirmed significant upregulation of HPSE, PCLAF, and SPRR2C mRNA levels in M5-induced psoriasis cell models. HPSE emerged as a multifunctional core gene associated with immune dysregulation. Molecular docking additionally predicted a potential interaction between the environmental pollutant BPA and HPSE; however, this interaction remains a computational hypothesis requiring experimental validation.

## Graphical Abstract

**Figure:**
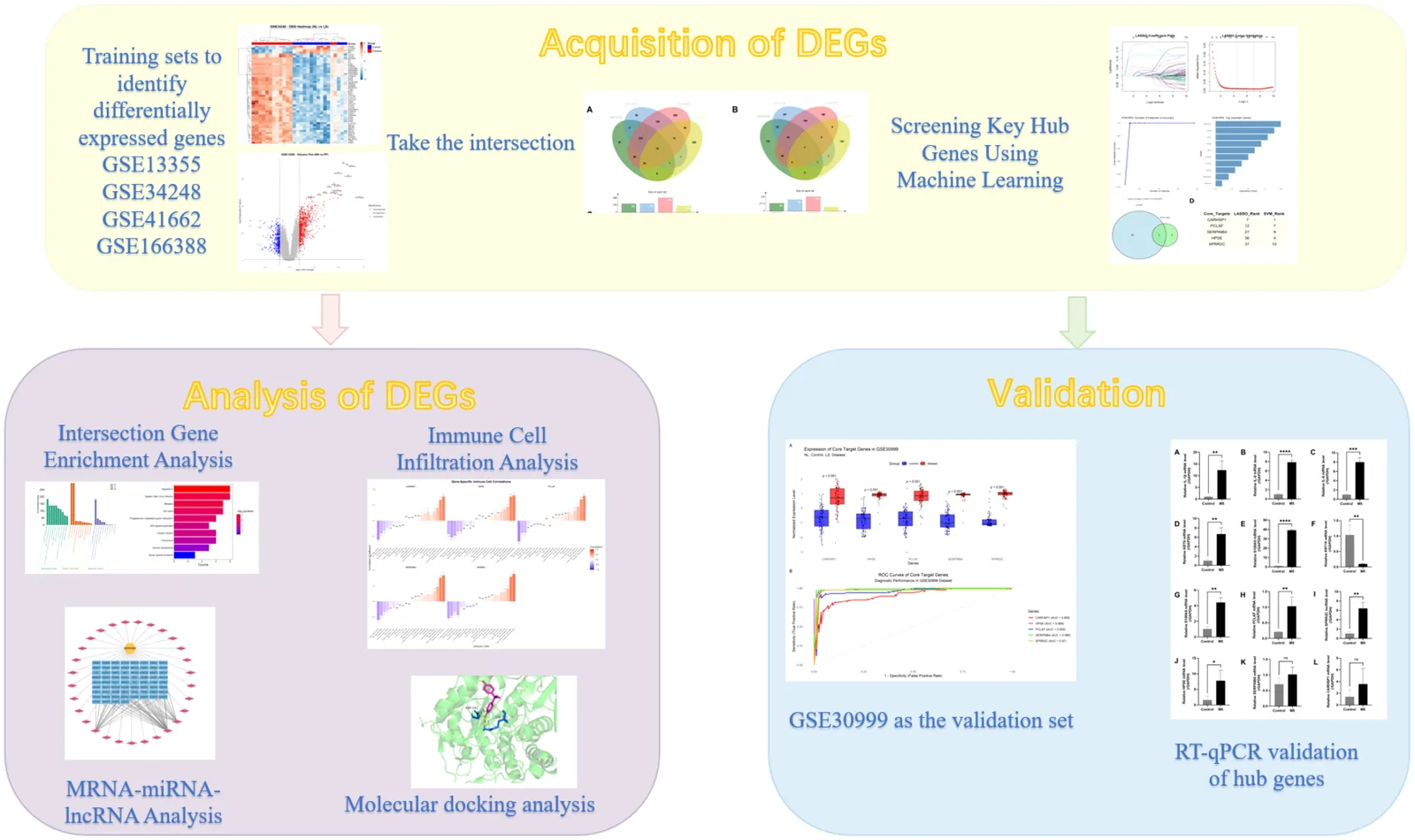

## Introduction

Psoriasis(PSO)is an immune-mediated, genetic disease manifesting in the skin or joints or both (1). Characterised by persistent inflammation and excessive proliferation of skin cells (2), it occurs worldwide and can affect individuals at any age, imposing a significant burden upon patients and society (3). Psoriasis encompasses multiple subtypes, including plaque, guttate, pustular, and erythrodermic forms, with plaque psoriasis being the most prevalent (4). These subtypes may coexist within the same individual; although they share similar pathogenic mechanisms, each variant involves distinct molecular and immune pathways (5). Common patient symptoms include pain, itching, and bleeding (6). When psoriasis is widespread or affects functionally critical areas such as the face, palms, soles, and genitals, the impact on patients’ lives and psychological well-being is particularly pronounced (7). Although psoriasis affects patients’ health status and life expectancy, a cure for the disease has not yet been achieved (8). Several treatment options exist to alleviate skin symptoms. Topical therapies, phototherapy, oral systemic treatments, and biologic therapies constitute the standard choices for managing psoriasis (9).

Elevated levels of multiple known pro-inflammatory cytokines are present in the serum of psoriasis patients, including TNF-α, IFN-γ, IL-6, IL-8, IL-12, IL-17A, and IL-18 (10). Biologic agents targeting several key cytokines have been introduced into clinical practice (11). However, owing to psoriasis’s multi-targeted, multi-pathway pathogenesis, monotherapy biologics struggle to achieve optimal efficacy (12). Concurrently, these treatment regimens carry varying degrees of side effects, potentially diminishing patient satisfaction (13). Consequently, there is an urgent need to develop novel therapeutic targets and medications. We aimed to identify differentially expressed genes between psoriasis and healthy controls by analysing PSO-related integrated gene expression datasets (GEO). Subsequent analyses, including Gene Ontology enrichment, Kyoto Encyclopedia of Genes and Genomes pathway analysis and machine learning, predicted and validated five key genes and pathways associated with psoriasis. This may offer novel perspectives and therapeutic targets for psoriasis treatment.

## Materials and methods

### Data acquisition and study design

The microarray expression datasets GSE13355, GSE34248, GSE41662 and GSE166388 were downloaded from the Gene Expression Omnibus (GEO, https://www.ncbi.nlm.nih.gov/geo/) database as training sets for psoriasis. Of these, GSE13355 and GSE166388 compared psoriatic lesional skin with healthy normal skin (NN), whereas GSE34248 and GSE41662 compared psoriatic lesional skin with paired non-lesional skin (NL). Because these two comparison types address different aspects of psoriasis biology, the final gene set is interpreted as representing lesion-associated genes that are consistently overexpressed in psoriatic lesions across cohorts, rather than as genes specifically linked to disease initiation versus healthy skin. GSE30999 was employed as an independent validation set. This dataset contains paired lesional and non-lesional skin samples from 85 patients with moderate-to-severe psoriasis and does not include healthy control skin. Therefore, the validation analysis assesses the ability of the hub genes to distinguish psoriatic lesional skin from paired non-lesional skin within the same patients. Furthermore, to rigorously prevent data leakage, we systematically cross-checked the individual sample accession numbers (GSM IDs) and patient metadata between the validation set (GSE30999) and the training sets (GSE13355). We explicitly verified that there is no sample overlap (distinct GSM accession ranges and patient identifier formats), ensuring that the validation cohort is completely independent of the training cohorts. The detailed information show in **Table 1**.

**Table 1:** Detailed information of GEO accession.

GEO accession	Data type	Platform	Tissue type	Sample size (disease vs control)	Clinical characteristics	Role
GSE13355	Expression profiling by array	GPL570	Skin	58 vs 64	Psoriasis vs healthy normal skin	Training
GSE34248	Expression profiling by array	GPL570	Skin	14 vs 14	Psoriasis vs paired non-lesional skin	Training
GSE41662	Expression profiling by array	GPL570	Skin	24 vs 24	Psoriasis vs paired non-lesional skin	Training
GSE166388	Expression profiling by array	GPL570	Skin	4 vs 4	Psoriasis vs healthy control	Training
GSE30999	Expression profiling by array	GPL570	Skin	85 vs 85	Moderate-to-severe psoriasis vs non-lesional skin	Validation

### Acquisition of genes with differential expression

Notably, all four training datasets utilized the identical microarray platform (GPL570: Affymetrix Human Genome U133 Plus 2.0 Array), ensuring complete consistency in probe design and dynamic range. To avoid systematic biases, differential expression analysis was performed independently on each of the four training datasets rather than merging them into a single concatenated matrix. For each dataset, probe IDs were mapped to gene symbols using the unified GPL570 platform annotation file; in cases where multiple probes mapped to a single gene, the average expression value was calculated. For datasets with paired samples (GSE34248, GSE41662, and GSE30999), differential expression analysis was performed using limma with a paired design: patient identifiers were incorporated as a blocking factor using `duplicateCorrelation()` to model within-patient correlation, and the design matrix included patient ID and tissue type (lesional vs non-lesional) as the contrast of interest. For unpaired datasets (GSE13355 and GSE166388), a standard two-group design comparing lesional skin with healthy skin was used. Differentially expressed genes (DEGs) for each dataset were identified using the ‘limma’ package in R software, with screening criteria set at |logFC| > 1 and an adjusted P-value < 0.05.

Subsequently, the robust, common differentially expressed genes characterizing psoriasis were obtained by taking the strict intersection of the independent DEGs across all four datasets. Because random intra-dataset batch artifacts are highly unlikely to be conserved across four independent laboratory cohorts, this strict intersection strategy serves as a stringent filter to eliminate dataset-specific noise, thereby capturing the most reliable transcriptomic signatures.

### Intersection gene enrichment analysis

The obtained psoriasis intersection genes underwent enrichment analysis across Gene Ontology (GO) categories: Molecular Function (MF), Biological Process (BP), and Cellular Component (CC), using the R packages ‘org.Hs.eg.db’ and ‘clusterProfiler’. Additionally, Kyoto Encyclopedia of Genes and Genomes (KEGG) enrichment analysis was performed on the intersecting genes identified in psoriasis. An adjusted p-value < 0.05 served as the filtering criterion. Visualization was conducted via the Micro Bioinformatics website.

### Screening key hub genes using machine learning

Two machine learning algorithms—Least Absolute Shrinkage and Selection Operator (LASSO) regression and the SVM-RFE algorithm—were used to screen key hub genes among the 77 intersecting DEGs. For both algorithms, the dataset used was GSE30999, which contains paired lesional and non-lesional skin samples from 85 patients with moderate-to-severe psoriasis (170 samples in total). The input matrix consisted of the normalized expression values of the 77 intersecting DEGs across these 170 samples, so the number of input features was 77. LASSO regression was performed using the R package ‘glmnet’ with family = “binomial”; the outcome variable was coded as 1 for psoriatic lesional samples and 0 for non-lesional controls, and the binomial deviance was used as the loss function. The penalty parameter λ was determined through 5-fold cross-validation, and the lambda.1se value was selected to obtain a parsimonious set of features. SVM-RFE was performed using the caret package with a linear kernel; the cost parameter C was tuned over the grid {0.01, 0.1, 1, 10, 100} using 5-fold cross-validation; feature scaling was applied by centering and scaling each gene to zero mean and unit variance; the number of retained features was optimized by 5-fold cross-validation with one repeat and a fixed random seed (set.seed(1)). The intersection of genes selected by the two algorithms was then assessed by receiver operating characteristic (ROC) curve analysis on the same GSE30999 dataset. ROC curves were generated using the ‘pROC’ package (version 1.18.0) in R. The area under the curve (AUC) and its 95% confidence interval (CI) were calculated using the DeLong method (14) to evaluate the ability of each hub gene to distinguish psoriatic lesional skin from paired non-lesional skin. An AUC value > 0.7 was considered to indicate good discriminative potential. Because feature selection and ROC evaluation were performed on the same dataset, these results should be interpreted with caution. To assess stability, patient-level bootstrap resampling (1,000 iterations) was additionally performed, but the DeLong method was used as the primary approach for all reported AUC values and confidence intervals.

### Immune cell infiltration analysis

We selected the CIBERSORT infiltration analysis method to analyze the psoriasis training dataset. CIBERSORT is a deconvolution method based on the principle of linear support vectors. For expression matrices of 22 human immune cell subtypes, it converts gene expression matrices into immune cell infiltration matrices. Gene expression data for 22 immune cell types and the CIBERSORT.R package were downloaded from the official CIBERSORT website (https://cibersort.stanford.edu/). Immune cell infiltration was analyzed using R software to obtain the expression matrix of immune cells from suitable samples under screening conditions, with a threshold of P < 0.05. The analysis yielded immune infiltration results to determine differences in immune cell infiltration between the psoriasis group and the healthy control group.

### Analysis of immune-related properties of key hub genes

By examining the correlation between key hub gene expression and immune cell content, this study investigates the influence of hub genes on immune cell infiltration, using P < 0.05 as the screening criterion.

### MRNA-miRNA-lncRNA analysis

MiRNAs targeting hub genes were identified from three databases: miRanda, miRDB, and TargetScan. A score of 1 was assigned when a gene matched a relevant miRNA in any corresponding database. A score of 3 was assigned upon matching all three databases. MiRNA-lncRNA interactions were mapped using the SpongeScan database. These non-coding RNAs were intersected with hub genes via Cytoscape software to construct mRNA-miRNA-lncRNA gene networks.

### Relationship between the environmental pollutant bisphenol A and key hub genes

The chemical structure and Simplified Molecular Input Line Entry System (SMILES) notation for bisphenol A (BPA) were retrieved from the PubChem database (https://pubchem.ncbi.nlm.nih.gov/). Species were designated as ‘Homo sapiens’ via ChEMBL (https://www.ebi.ac.uk/chembl/), STITCH (http://stitch.embl.de/), and the Swiss-Target Prediction database (http://swisstargetprediction.ch/). Targets were merged and duplicates removed. Psoriasis-associated disease targets were sourced from the Online Mendelian Inheritance in Man (OMIM) (https://omim.org/) and GeneCards (https://www.genecards.org/) databases. Intersections were formed between BPA, psoriasis, and the aforementioned hub genes. Molecular docking analysis was performed between BPA and the resulting target intersections, calculating hydrogen bonds and binding energies.

### Therapeutic agents

Existing therapeutic agents for psoriasis were investigated via the MalaCards website (https://www.malacards.org/). The DGIdb website (https://www.dgidb.org/) was employed to predict drugs based on the hub genes. Molecular docking was then used to combine the hub genes with the predicted drugs, calculating binding energies and hydrogen bond counts, with visualisation achieved using pymol.

### Cell culture and processing

The HaCaT cell line was purchased from the Shanghai Institute of Biological Sciences, Chinese Academy of Sciences (Shanghai, China). Interleukin-17A (IL-17A), Interleukin-1α (IL-1α), Interleukin-22 (IL-22), and Oncostatin M (OSM) were purchased from ACRO Biosystems (Delaware). Recombinant human tumour necrosis factor-α (TNF-α) was purchased from Peprotech (New Jersey). HaCaT cells were seeded at a density of 2 × 10^5^ cells per well in 6-well plates, with 2 ml of Dulbecco’s Modified Eagle’s Medium supplemented with 10% foetal bovine serum and 1% penicillin-streptomycin added to each well. Cells were cultured at 37 °C in humidified 5% CO_2_ until confluence reached 60% to 70%. Cells were then stimulated for 12 hours with medium containing M5 (IL-17A, IL-22, Oncostatin M, IL-1α, and TNF-α at a final concentration of 10 ng/ml) to mimic the hyperproliferative characteristics of psoriatic keratinocytes for *in vitro* experiments.

### RNA extraction and cDNA synthesis

Total RNA was extracted from M5-treated HaCaT cells using the RNA Quick Purification Kit (ES Science, Shanghai, China). RNA concentration and quality were assessed using Thermo Fisher Scientific NanoDrop. RNA was reverse transcribed into cDNA using the Fast All-in-One RT Kit (with gDNA Remover) (ES Science, Shanghai, China).

### Real-time quantitative PCR

Real-time fluorescent quantitative PCR (RT-qPCR) was employed to detect predicted psoriasis-associated genes and cytokine genes in naïve HaCaT cells (without M5 stimulation, negative control) and M5 (10 ng/mL)-stimulated HaCaT cells (psoriasis cell model). RT-qPCR was performed according to the protocol for ES Science 2× Super SYBR Green qPCR Master Mix. Primer sets for selected genes were designed by Sangon Biotech (Shanghai, China). Primer sequences for PCR are shown in **Table 2**. Expression data were normalized using the reference gene glyceraldehyde-3-phosphate dehydrogenase (GAPDH). mRNA levels were calculated using the 2−ΔΔCt method.

**Table 2:** PCR primer for quantitative real-time PCR confirmation of hub genes.

Gene	Primer sequence (5′ → 3′)	Reverse sequence (3′ → 5′)
GAPDH	F: CAGGAGGCATTGATGATGAT	R: GAAGGCTGGGGCTCATTT
IL-1β	F: AGCTACGAATCTCCGACCAC	R: CGTTATCCCATGTGTCGAAGAA
IL-6	F: CCTGAACCTTCCAAAGATGGC	R: TTCACCAGGCAAGTCTCCTCA
IL-8	F: GTCCTTGTTCCACTGTGCCT	R: GCTTCCACATGTCCTCACAA
S100A8	F: CAAGGAAATCACCATGCCCTCTA	R: ACCATCGCAAGGAACTCCTCGA
S100A9	F: TCATCAACACCTTCCACCAATAC	R: ATCTTTTCGCACCAGCTCTTT
KRT6A	F: ATGGCAGCCCTGGACAAG	R: TCAGTGGTGATGCGGATGTA
KRT10	F: CCTGCTTCAGATCGACAATGCC	R: ATCTCCAGGTCAGCCTTGGTCA
HPSE	F: GACTTCTTCACCCAGGAGCC	R:ACCAAACCTCAGGTACGCAG
SPRR2C	F: TCCTTTCTGAGGCTGCCATG	R: AGAAGCTCATGCCAGGTGAC
PCLAF	F: TGTCCAGTGAAACACCCTCG	R: TGCTTTAGTCCGCACCATGT
SERPINB4	F: CAGACCTCTCAGGCATGACC	R: ACTCCCTCCTCAGTGACCTC
CARHSP1	F: CCCCTCTTAGAGCCTGACCT	R: GTCCTCTCCACAAGCCCTTC

### Statistical analysis

Pearson correlation analysis was employed to determine the association between hub genes and immune cell expression, with analyses conducted within the R programming environment. Relative expression levels of five hub genes and seven cytokine genes in HaCaT cells subjected to different treatments were compared using GraphPad Prism 10.2.3. All RT-qPCR experiments were performed with three independent biological replicates, each assayed with two technical replicates. Data were compared using unpaired t-tests with Welch’s correction; for comparisons involving multiple genes, P values were adjusted using the Benjamini-Hochberg false discovery rate method. Results are presented as mean ± standard deviation, and statistical significance was set at P < 0.05.

## Results

### Differential expression analysis of psoriasis-associated genes

We selected GSE13355, GSE34248, GSE41662, and GSE166388 as training datasets for psoriasis. Differential analysis was performed on skin tissue from psoriasis patients and healthy controls, using |logFC| > 1 and an adjusted P-value < 0.05 as screening criteria. The number of differentially upregulated genes was 505, 526, 868, and 384, respectively; Downregulated differentially expressed genes numbered 292, 428, 543, and 150 respectively. The results of differentially expressed genes in four training sets show in **Figure 1**. Intersecting the up- and downregulated differentially expressed genes across the four datasets yielded 70 intersecting up-regulated genes and 7 intersecting down-regulated genes, show in **Figures 2A, B**.

**Figure 1:**
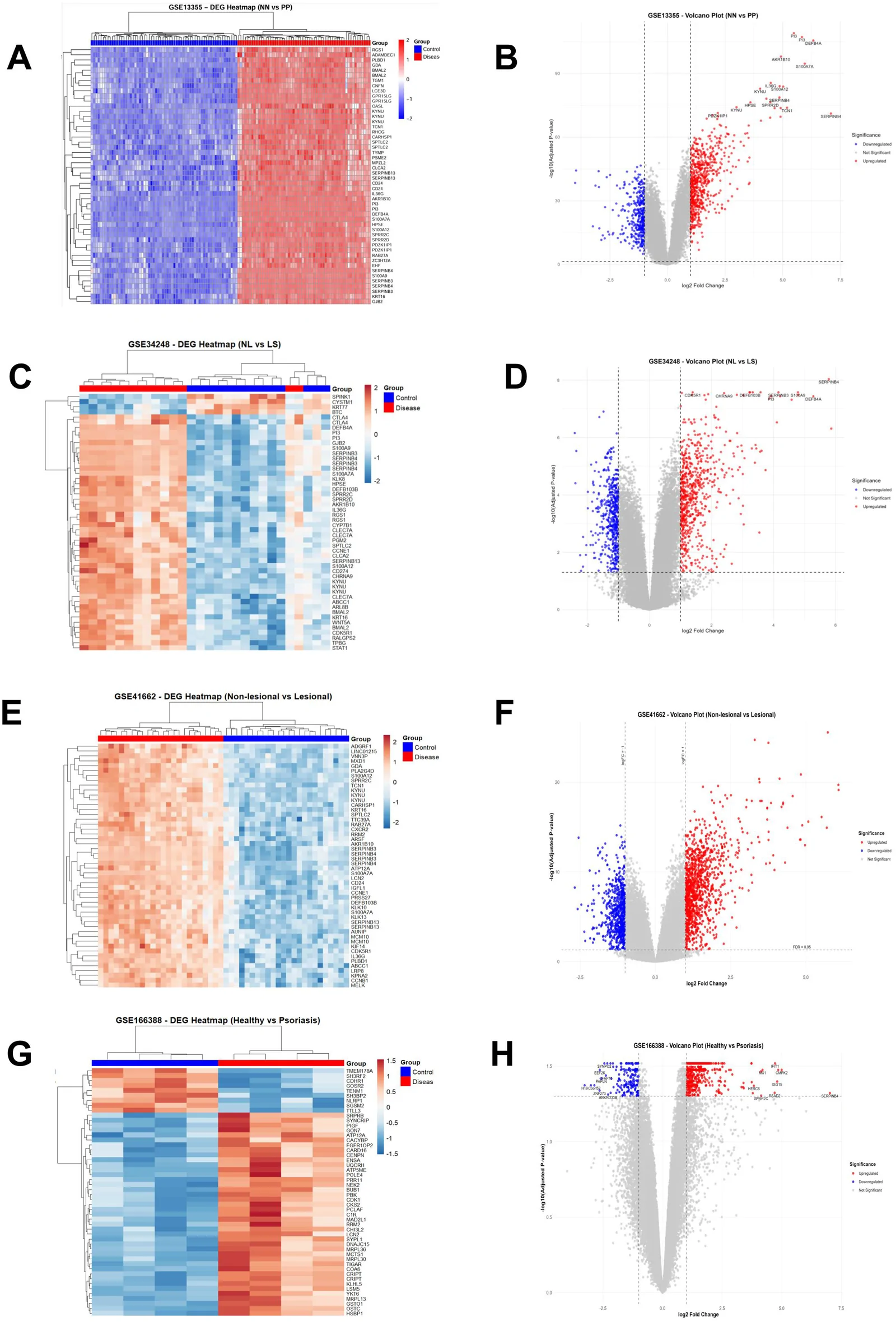
Differentially expressed genes in psoriasis. **(A)** Heatmap of the differentially expressed genes in GSE13355; **(B)** volcano map of the differentially expressed genes in GSE13355; **(C)** Heatmap of the differentially expressed gene in GSE34248; **(D)** volcano map of the differentially expressed genes in GSE34248; **(E)** Heatmap of the differentially expressed gene in GSE41662, **(F)** volcano map of the differentially expressed genes in GSE41662; **(G)** Heatmap of the differentially expressed gene in GSE166388, **(H)** volcano map of the differentially expressed genes in GSE166388.

**Figure 2:**
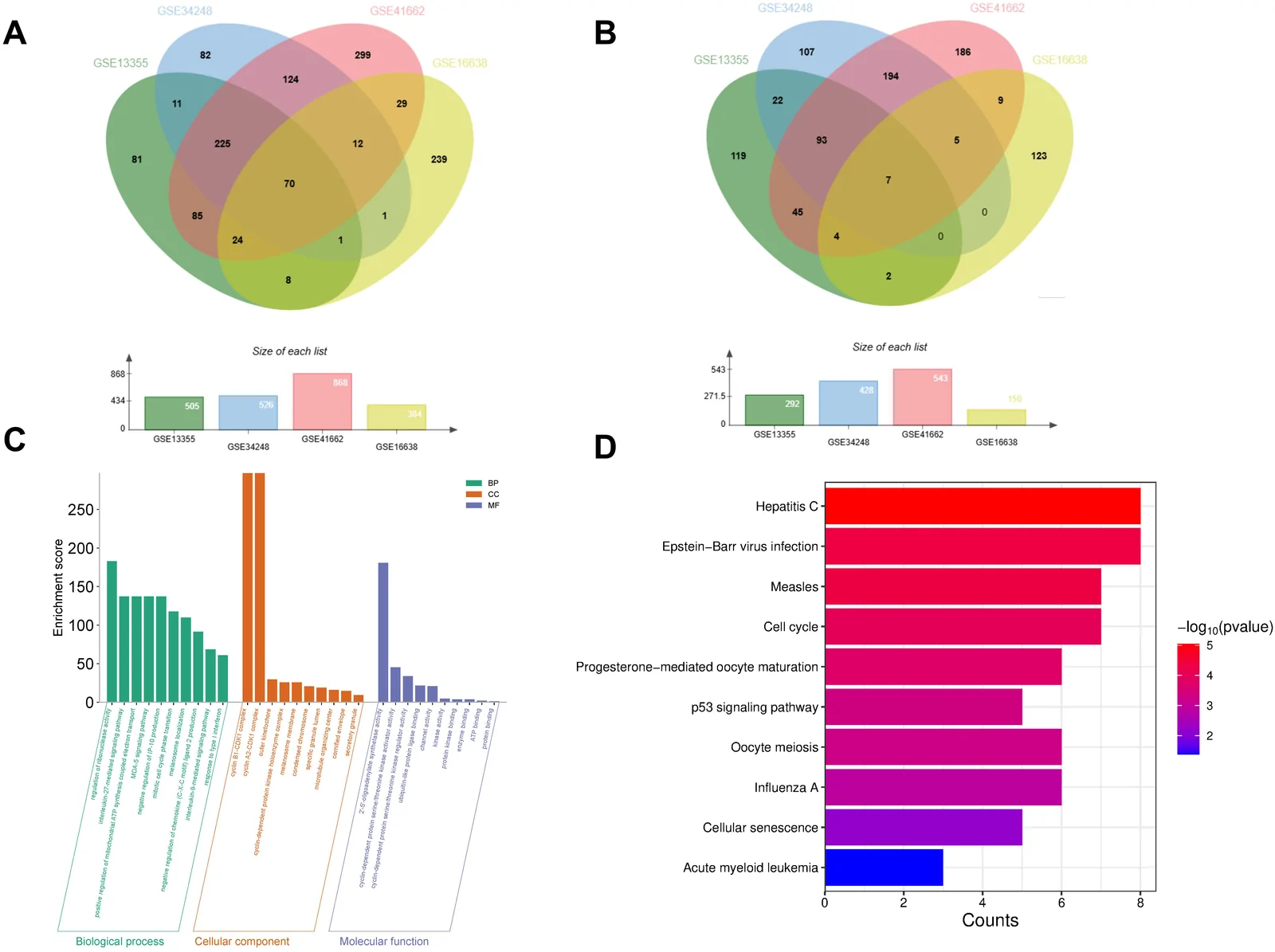
Enrichment analysis. **(A)** Venn diagram of differentially expressed genes upregulated in the psoriasis training set; **(B)** Venn diagram of differentially expressed genes downregulated in the psoriasis training set; **(C)** Gene Ontology (GO) enrichment analysis; **(D)** KEGG analysis of enrichment.

### Intersection target enrichment analysis

We conducted enrichment analysis on the selected overlapping target genes to explore their potential biological functions and regulatory mechanisms. Analysis of GO enrichment results across three ontologies revealed that in Biological Processes (BP), enriched entries primarily centered on fundamental biological processes such as gene transcription regulation, cellular metabolism, immune response, and cell cycle regulation. At the Cellular Component (CC) level, enriched entries involved multiple cellular structures including the nucleus, endoplasmic reticulum, cell membrane, and clathrinid-coated vesicle membrane. Regarding molecular functions (MF), the enrichment primarily encompassed molecular binding and activity functions such as ATPase activity, RNA binding, protein receptor binding, and immunoglobulin binding. KEGG enrichment analysis revealed significant enrichment of pathways including hepatitis C, Epstein-Barr virus infection, measles, cell cycle, p53 signalling pathway, and oocyte maturation in the studied gene set. This suggests these genes may exert biological functions through involvement in pathways such as viral infection, cell proliferation, and apoptosis regulation. Show in **Figures 2C, D**.

### Machine learning analysis of hub targets

We employed the LASSO regression model and SVM-RFE method to screen key genes using the 77 intersecting DEGs as input features and GSE30999 as the dataset. In the LASSO regression analysis, 44 genes including KLK8, CMAHP, CDHR1, SYNCRIP, SLC16A1, HERC6, CARHSP1 were identified as characteristic genes. Concurrently, the SVM-RFE analysis selected ten genes CARHSP1, CD24, CKS2, HPSE, IRF7, KIF20A, PCLAF, RRM2, SERPINB4, and SPRR2C as characteristic genes. Merging and taking the intersection yielded five characteristic genes: CARHSP1, HPSE, PCLAF, SERPINB4, and SPRR2C. Show in **Figure 3**.

**Figure 3:**
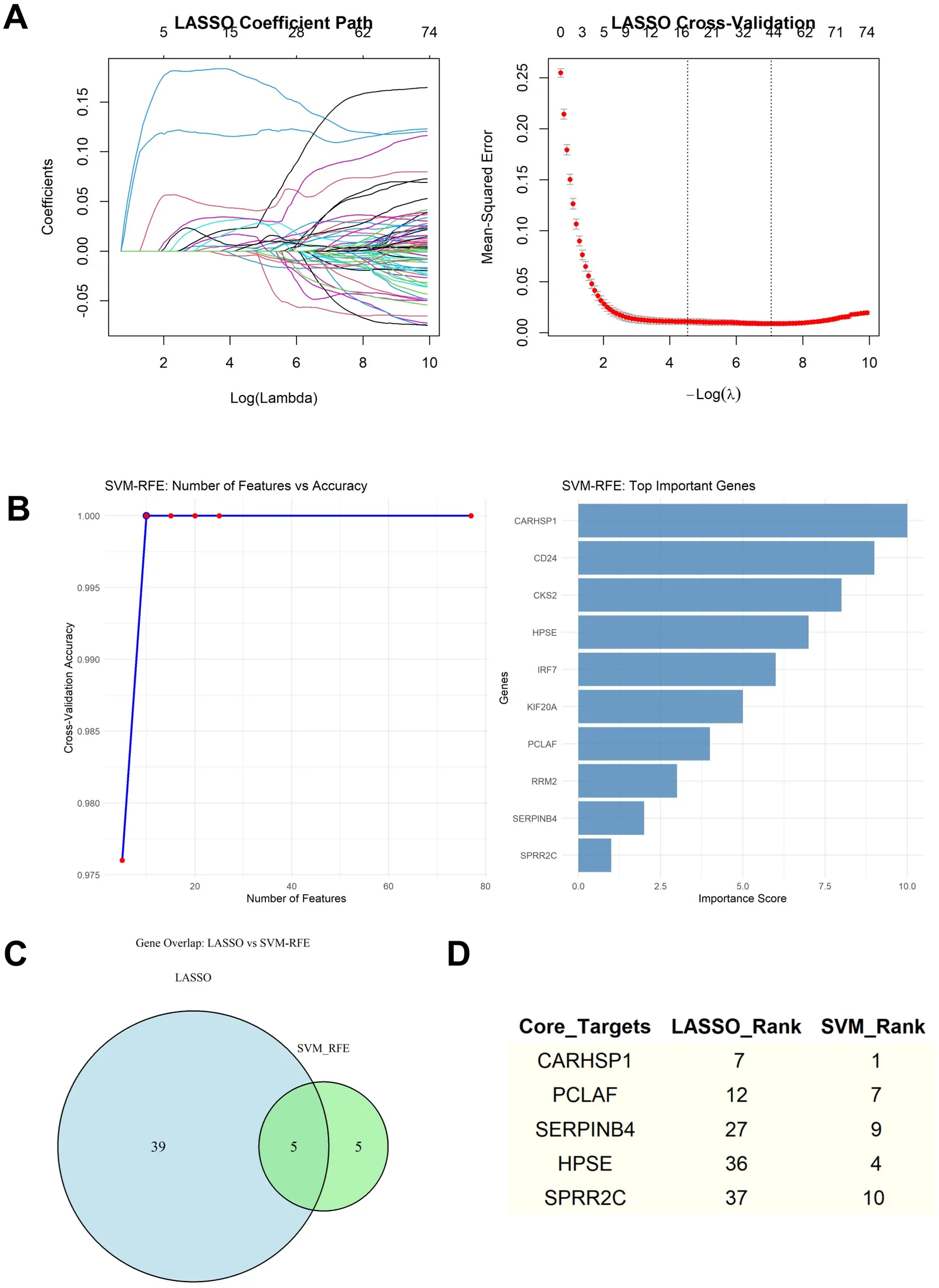
Two methods of screening the characteristic Hub genes. **(A)** LASSO coefficient paths and cross validation for psoriasis; **(B)** SVM-RFE Number of Features vs Accuracy and Scores of Top Important Genes for psoriasis; **(C)** Gene Overlap Venn Diagram between LASSO and SVM-RFE; **(D)** Ranking Comparison Table for Hub Targets.

### Validation of hub genes

We obtained the validation dataset GSE30999, which contains paired lesional and non-lesional skin samples from 85 patients with moderate-to-severe psoriasis. Receiver operating characteristic (ROC) curves were plotted, and area under the curve (AUC) was calculated to assess the ability of each hub gene to distinguish lesional from paired non-lesional skin. Compared with paired non-lesional skin, CARHSP1, PCLAF, SERPINB4, HPSE and SPRR2C were significantly upregulated in psoriatic lesional skin (p<0.05), with AUC values exceeding 0.9 for all. These findings demonstrate that the five hub genes have strong discriminative power for distinguishing psoriatic lesional skin from paired non-lesional skin in patients with moderate-to-severe psoriasis. This should not be interpreted as diagnostic performance for differentiating psoriasis from healthy skin or from other inflammatory dermatoses. However, as discussed below, the exclusive use of extreme phenotypes in this validation cohort may inflate the diagnostic metrics, and their utility for broader clinical applications requires further evaluation. Show in **Figure 4**. To address issues such as the risk of overfitting, we performed guided resampling (1,000 iterations) on the validation set GSE30999 to estimate the 95% confidence intervals for the AUC values of the five core genes. The results are as follows: CARHSP1(AUC 0.973; 95%CI: 0.943 – 0.995); HPSE (AUC 0.986; 95%CI: 0.965 – 0.998); PCLAF (AUC 0.948; 95%CI: 0.912 – 0.979); SERPINB4(AUC 0.982; 95%CI: 0.956 – 1.000); SPRR2C (AUC 0.970; 95%CI: 0.936 – 0.998). The bootstrap-based estimates were consistent with the DeLong estimates. Narrow confidence intervals indicate that the AUC estimates are statistically stable under resampling, but they do not exclude potential bias from feature selection or information leakage. Therefore, these AUC values should be interpreted as measures of separation between lesional and non-lesional skin in this specific cohort, rather than as evidence against overfitting.

**Figure 4:**
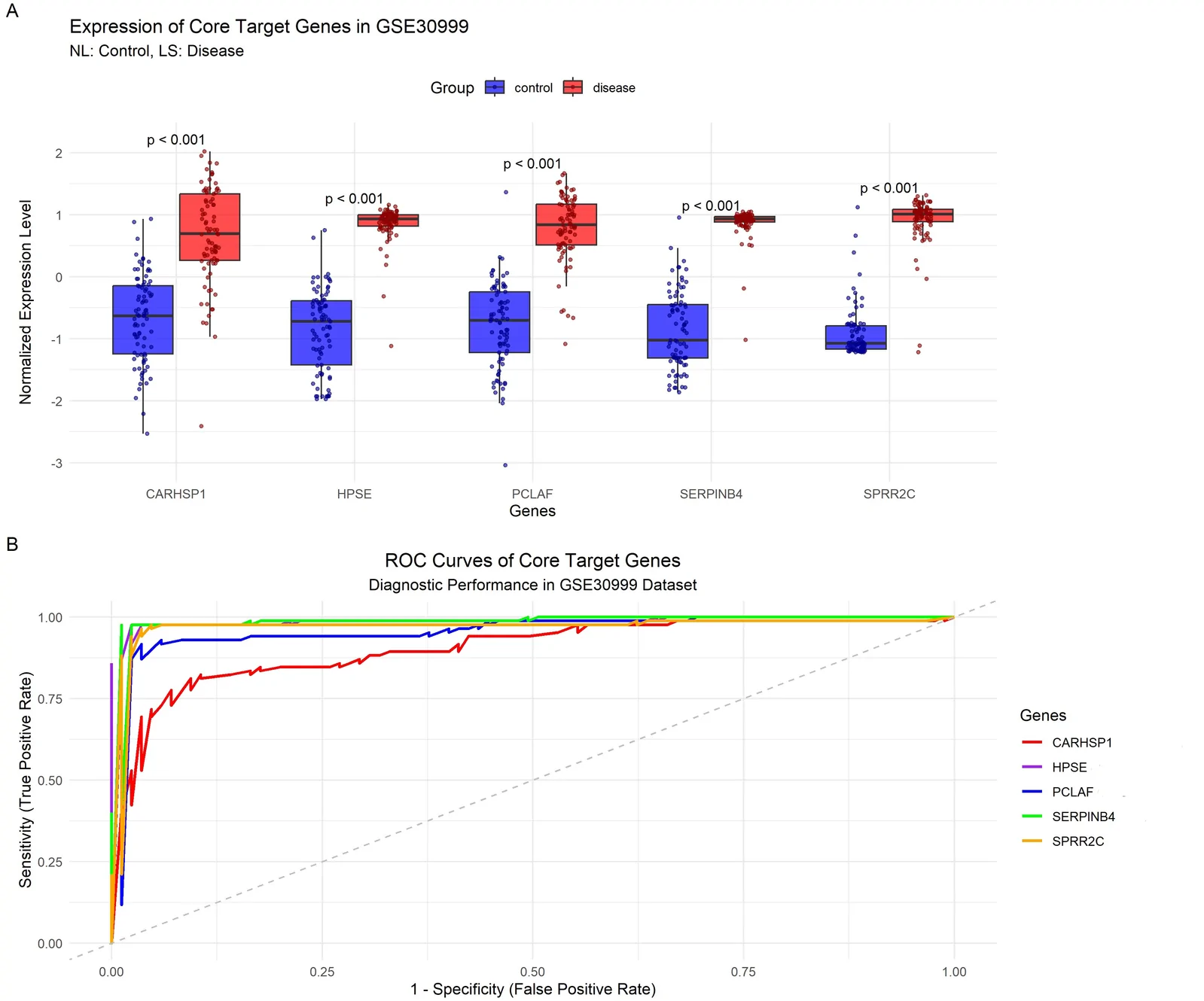
GSE30999 hub gene differential expression boxplots and ROC curve. **(A)** Boxplots showing the expression levels of five hub genes (CARHSP1, HPSE, PCLAF, SERPINB4, SPRR2C) between control and disease groups (p < 0.001). **(B)** ROC curves assess the discriminative ability of each hub gene to distinguish psoriatic lesional skin from paired non-lesional skin, these AUC values should not be interpreted as diagnostic performance for differentiating psoriasis from healthy skin or other inflammatory dermatoses.

### Predicted mRNA-miRNA-lncRNA regulatory network

Messenger RNA (mRNA) serves as the core of functional execution, with its encoded proteins directly participating in physiological/pathological processes (such as immune responses, cell proliferation, metabolic regulation, etc.). Micro RNA (miRNA) acts as the ‘regulatory executor’, suppressing mRNA expression by targeting the 3’UTR region to inhibit translation or promote degradation. Long non-coding RNA (lncRNA) functions as a ‘molecular sponge’, binding to miRNA to competitively release its inhibition on mRNA, thereby indirectly upregulating mRNA expression. In the pathogenesis of psoriasis, dysregulation of this network serves as a key driver. Therefore, elucidating the specific regulatory roles of the ‘mRNA-miRNA-lncRNA’ axis within the disease provides a ‘molecular interaction map’ for deciphering its mechanisms, while also identifying potential therapeutic targets and biomarkers. This study identified 119 miRNAs and 965 lncRNAs associated with HPSE; 73 miRNAs and 628 lncRNAs for CARHSP1; 42 miRNAs and 216 lncRNAs for PCLAF; 29 miRNAs and 77 lncRNAs for SERPINB4; while no corresponding miRNAs were detected for SPRR2C. Show in **Figure 5**. The mRNA-miRNA-lncRNA network was constructed solely from target prediction databases and was not filtered or validated using miRNA or lncRNA expression data from psoriatic tissues. The expected negative correlations between miRNAs and target mRNAs and positive correlations between lncRNAs and mRNAs were not examined. Consequently, the network represents predicted interactions only and does not establish a functional ceRNA mechanism. Future studies should integrate matched expression data to refine and validate these predicted interactions.

**Figure 5:**
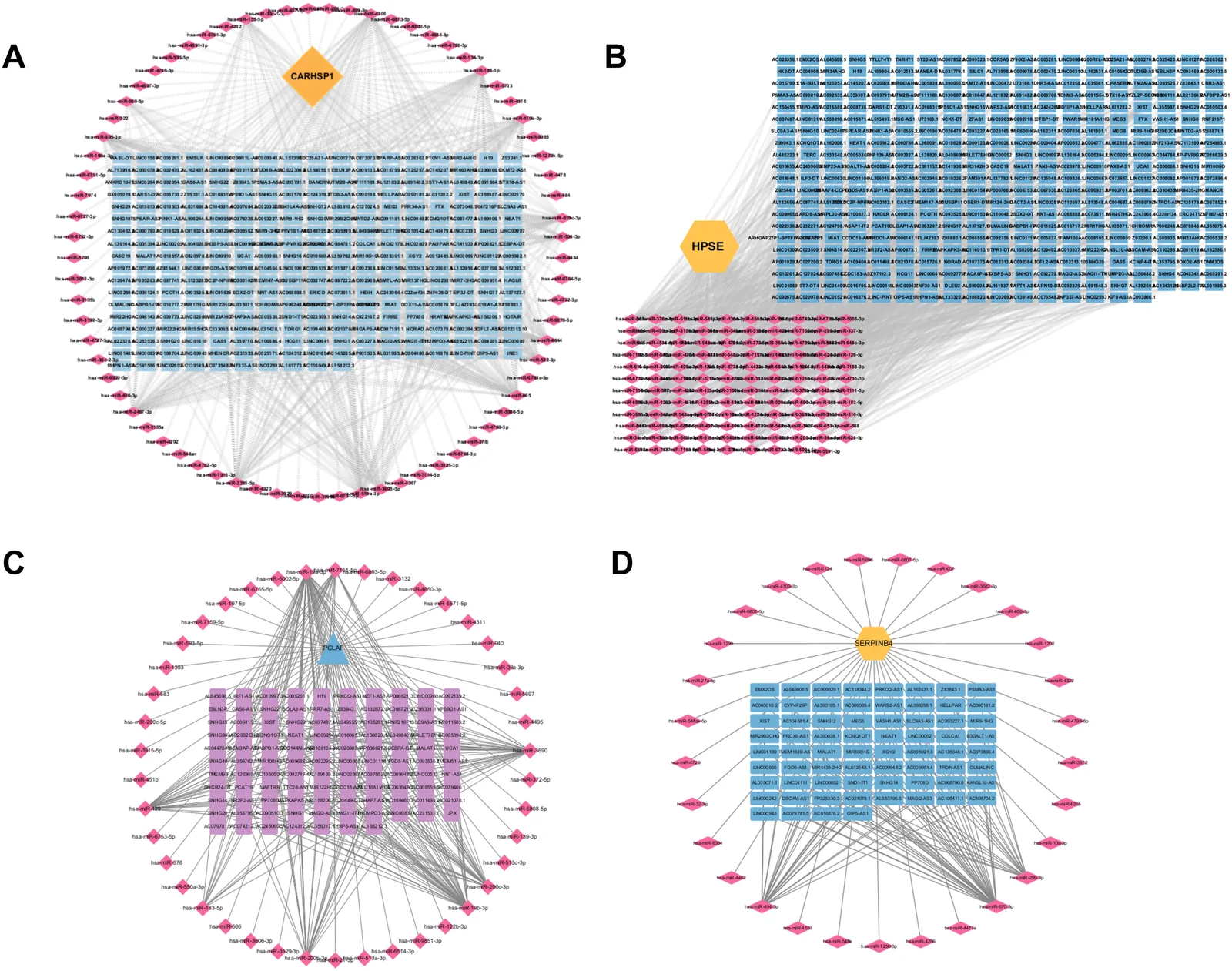
MRNA-miRNA-lncRNA network of hub gene. **(A)** Regulatory network centered on CARHSP1. **(B)** Regulatory network centered on HPSE. **(C)** Regulatory network centered on PCLAF. **(D)** Regulatory network centered on SERPINB4.

### Analysis of immune cell infiltration

Analysis of immune cell infiltration using the CIBERSORT method revealed increased infiltration of CD4+ memory activated T cells, γδ T cells, resting natural killer (NK) cells, M1 macrophages, and activated dendritic cells within psoriatic lesions. Conversely, infiltration of memory B cells, plasma cells, activated NK cells, and resting mast cells was diminished. Show in **Figure 6**.

**Figure 6:**
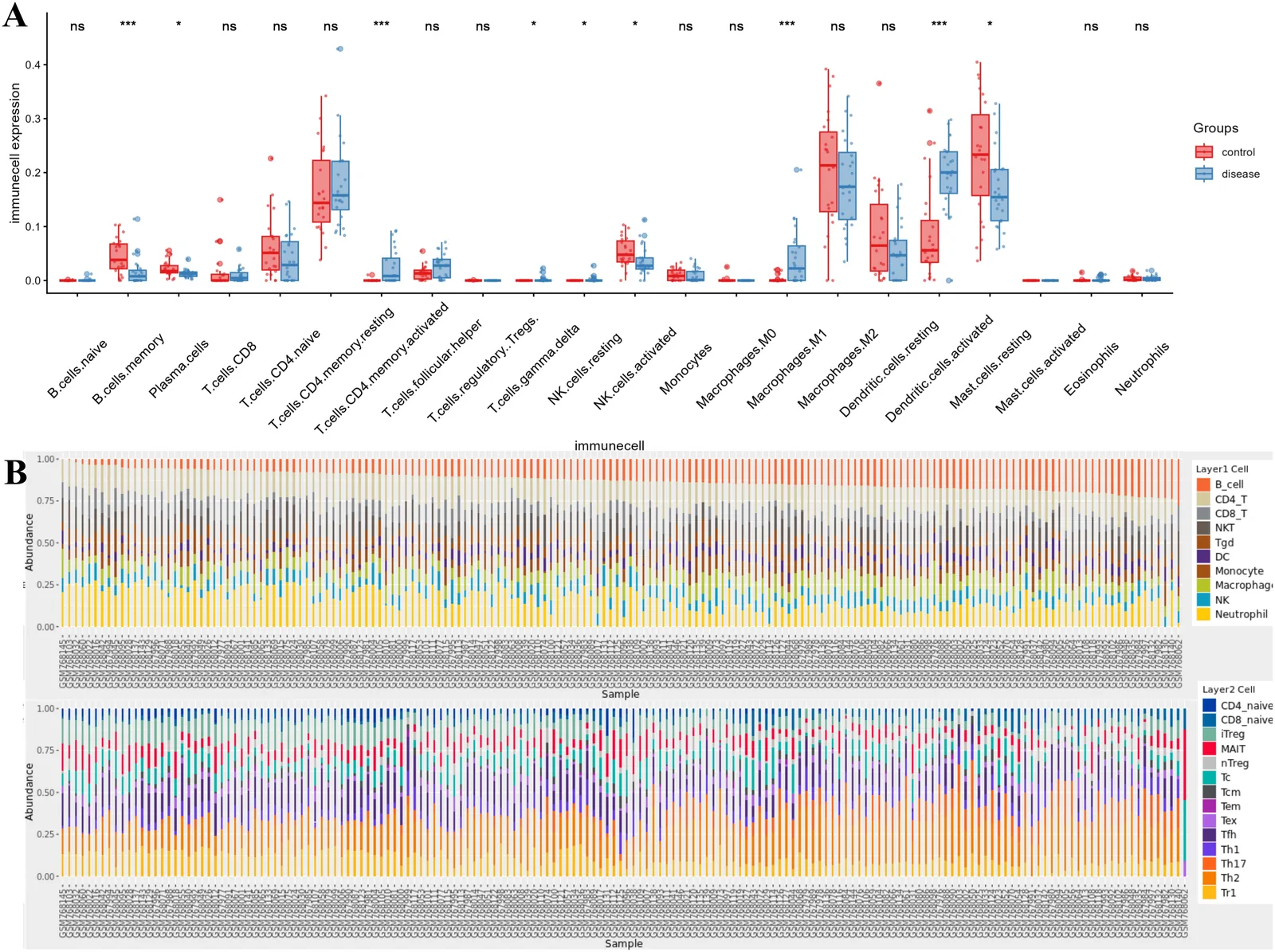
Results of immune cell infiltration analysis. **(A)** Boxplots comparing the expression of 22 immune cell types between control (red) and disease (blue) groups. Significance levels are indicated (*p < 0.05, ***p < 0.001; ns, not significant). **(B)** Stacked bar charts showing the relative abundance of immune cells across individual samples. The top panel displays Layer 1 (major cell types), and the bottom panel displays Layer 2 (detailed subtypes).

### Immune infiltration correlation of core genes

To control for false positives resulting from multiple comparisons, we applied the Benjamini-Hochberg False Discovery Rate (FDR) correction to the P-values of the correlations between the five core genes and the scores of 22 types of immune cell infiltration, using q < 0.05 as the significance threshold. Among all 110 tests, 56 associations were significant at the unadjusted P < 0.05 level, and 47 associations remained significant (q < 0.05) after FDR correction. The number and directional distribution of significant associations for each gene are as follows: CARHSP1 (10, 6 positive and 4 negative), HPSE (10, 5 positive and 5 negative), PCLAF (8, 5 positive and 3 negative), SERPINB4 (10, 6 positive and 4 negative), and SPRR2C (9, 5 positive and 4 negative). See **Figure 7** for specific association patterns.

**Figure 7:**
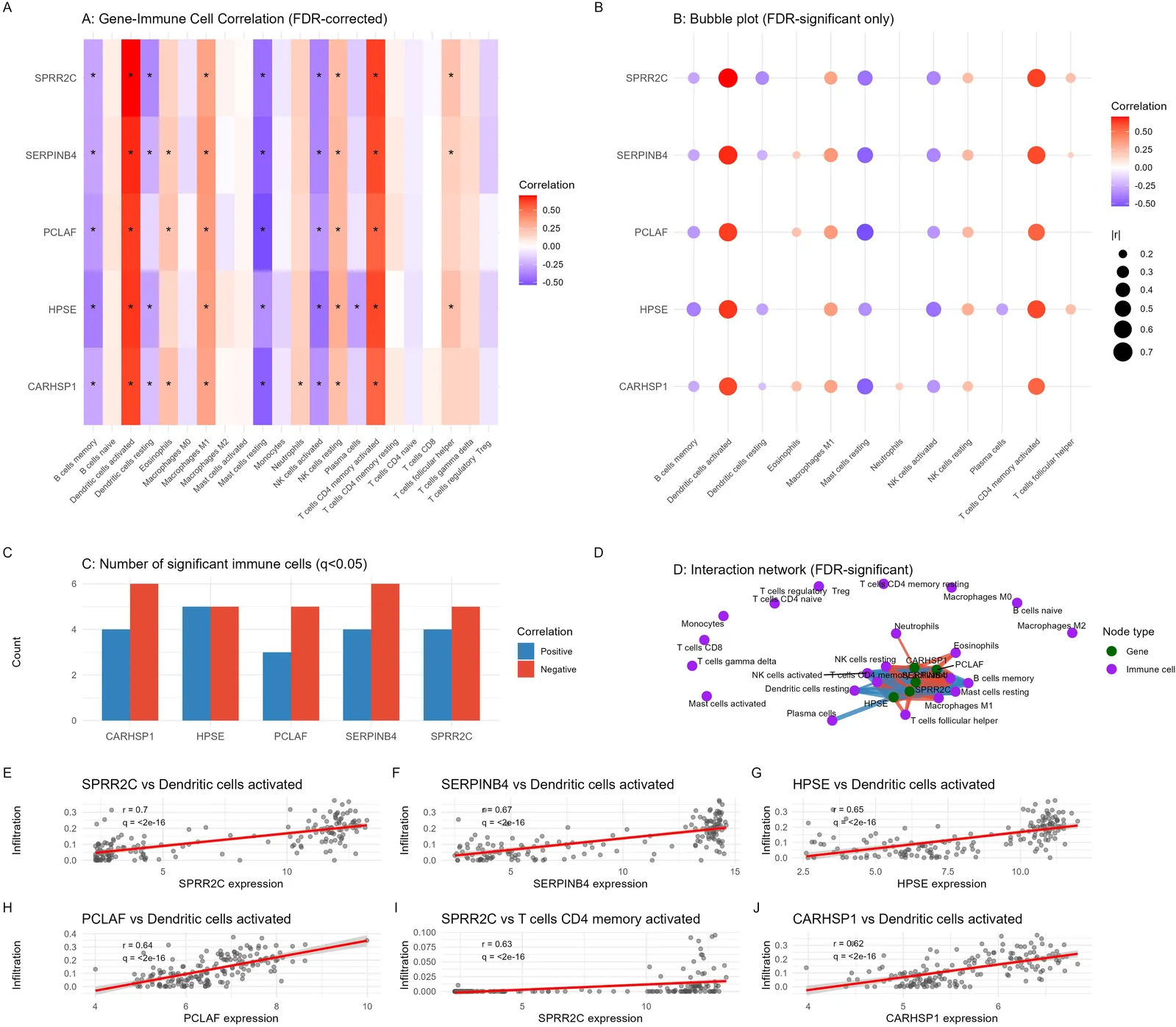
Analysis of immune cell infiltration-related hub genes. **(A)** Gene-Immune Cell Correlation *p < 0.05; **(B)** Bubble plot (FDR-significant only); **(C)** Number of significant immune cells (q<0.05); **(D)** Interaction network (FDR-significant); (**E–J)** Scatter plots with correlation.

CARHSP1 showed a total of 10 significant associations, including 6 positive correlations and 4 negative correlations. Its most significant positive correlations were observed with activated dendritic cells (r = 0.617, q = 5.56×10^^−^18^), activated CD4^+^ memory T cells (r = 0.534, q = 6.16×10^^−^13^), and M1 macrophages (r = 0.336, q = 3.49×10^^−^5^); while the strongest negative correlations were observed with resting mast cells (r = -0.488, q = 1.41×10^^−^10^) and activated NK cells (r = -0.331, q = 4.53×10^^−^5^).

HPSE also showed 10 significant associations, with 5 positive and 5 negative correlations. The strongest positive correlations were observed with activated dendritic cells (r = 0.648, q = 4.47×10^^−^20^), activated CD4^+^ memory T cells (r = 0.612, q = 1.24×10^^−^17^), and M1 macrophages (r = 0.361, q = 6.86×10^^−^6^); Negative correlations were primarily observed with activated NK cells (r = −0.432, q = 3.10 × 10^−8^), resting mast cells (r = −0.346, q = 1.87 × 10^−5^), and memory B cells (r = −0.390, q = 1.00 × 10^−6^).

PCLAF showed 8 significant associations, including 5 positive correlations and 3 negative correlations. The targets with the strongest positive correlations were activated dendritic cells (r = 0.636, q = 2.97 × 10^−^¹^9^), activated CD4^+^ memory T cells (r = 0.542, q = 2.96×10^−^¹³), and M1 macrophages (r = 0.364, q = 5.83×10^−6^); Negative correlations were most pronounced for resting mast cells (r = −0.535, q = 5.92 × 10^−13^) and activated NK cells (r = −0.317, q = 1.01 × 10^−4^).

SERPINB4 exhibited a bidirectional association pattern, with 6 of the 10 significant associations being positive and 4 being negative. The strongest positive correlations were observed with activated dendritic cells (r = 0.667, q = 1.80 × 10^−^²¹), activated CD4^+^ memory T cells (r = 0.605, q = 3.19×10^−17^), and M1 macrophages (r = 0.379, q = 2.18×10^−6^); Negative correlations were primarily observed with resting mast cells (r = −0.478, q = 3.77 × 10^−10^), activated NK cells (r = −0.372, q = 3.48 × 10^−6^), and resting dendritic cells (r = −0.240, q = 0.0046).

SPRR2C also exhibited bidirectional associations, with 5 of the 9 significant correlations being positive and 4 being negative. Its strongest positive correlations were observed with activated dendritic cells (r = 0.703, q = 1.27 × 10^−^²^4^), activated CD4^+^ memory T cells (r = 0.627, q = 1.22 × 10^−^¹^8^), and M1 macrophages (r = 0.334, q = 3.79 × 10^−5^); Negative correlations were primarily observed with activated NK cells (r = −0.373, q = 3.38 × 10^−6^), resting mast cells (r = −0.431, q = 3.20 × 10^−8^), and resting dendritic cells (r = −0.358, q = 8.15 × 10^−6^).

Overall, all five core genes showed significant positive correlations with activated dendritic cells, activated CD4^+^ memory T cells, and M1 macrophages, while exhibiting significant negative correlations with resting mast cells, activated NK cells, and others. Among these, HPSE and PCLAF exhibited the broadest correlations with various T-cell subsets and myeloid cells; CARHSP1 was primarily positively correlated with activated immune cells, suggesting it may be involved in promoting immune activation; whereas SERPINB4 and SPRR2C displayed a typical bidirectional association pattern, showing positive correlations with activated cells and negative correlations with certain quiescent/cytotoxic cells. These results suggest that the aforementioned genes may play multiple roles in the immune regulatory network of psoriasis. However, whether these correlations reflect direct biological interactions or are secondary consequences of the inflammatory microenvironment remains to be further determined by functional experiments.

### Pharmacological effects

The MalaCards website indicates that 394 existing psoriasis treatments are available, with 168 approved for clinical use. Thirty-nine are in Phase 4 (post-marketing studies). Furthermore, the DGIdb platform predicts drugs corresponding to core targets, with HPSE associated with 18 predicted compounds. No corresponding drugs were identified for the remaining hub genes. Molecular docking was performed with the top three drugs exhibiting highest interaction scores against HPSE. While DGIdb predicted the general class of antiestrogens, the specific representative compound Genistein (PubChem CID: 5280961) was selected and downloaded for the molecular docking simulation to provide an accurate 3D ligand structure. Results indicate a strong binding affinity between HPSE and Genistein, characterized by the highest number of hydrogen bonds, lowest binding energy, and stable conformations of both ligand and receptor. Show in **Figures 8A–C**.

**Figure 8:**
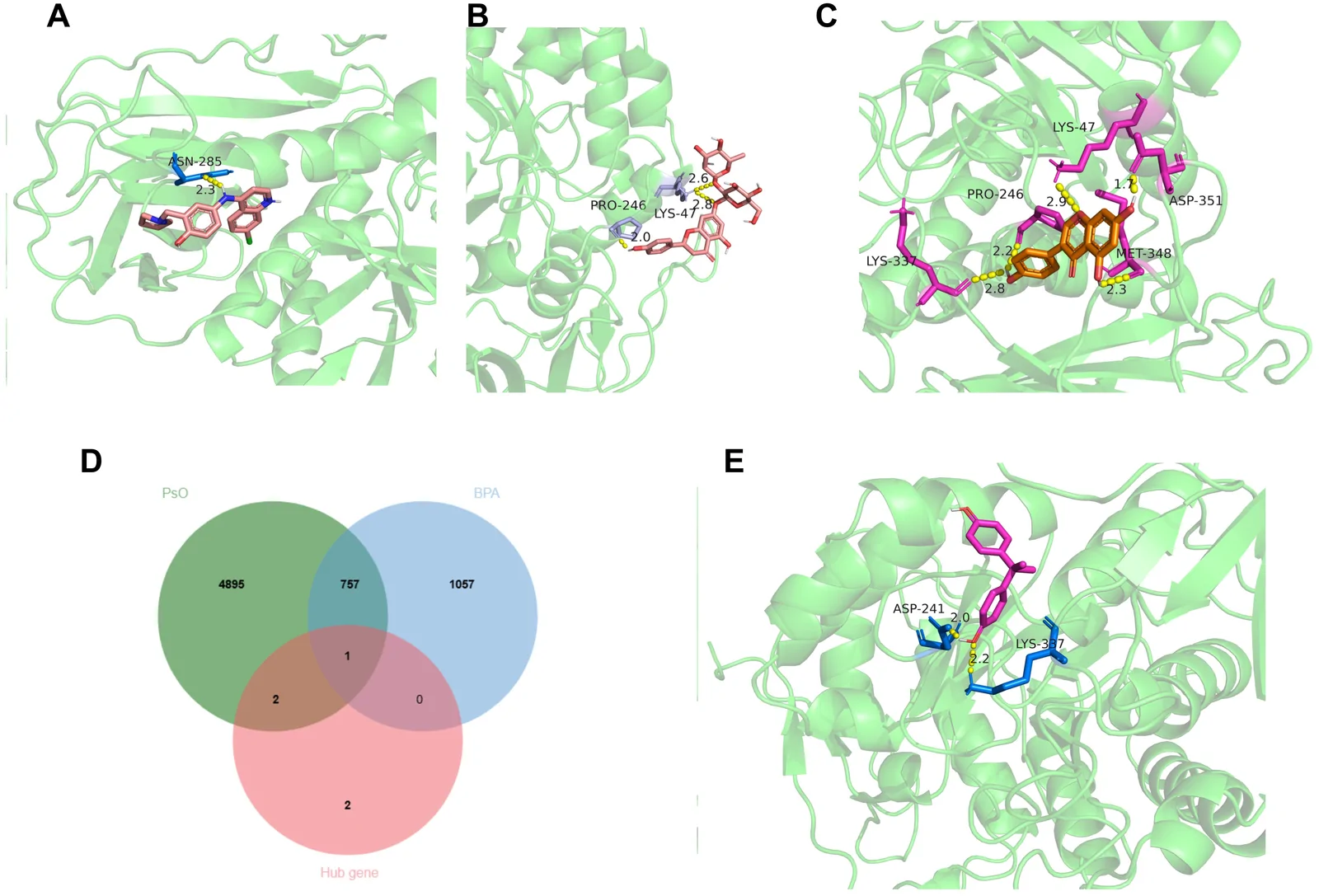
Molecular docking. **(A)** HPSE with AMOPYROQUINE; **(B)** HPSE with NARINGIN; **(C)** HPSE with Genistein; **(D)** Venny result of PSO, BPA and hub gene; **(E)** HPSE with BPA.

### The environmental endocrine disruptor bisphenol A (BPA) exerts pathogenic effects on psoriasis via HPSE

We conducted molecular docking analysis as part of a network toxicology framework to evaluate interactions between BPA and intersected target proteins. BPA exhibited a binding affinity with HPSE, with a binding energy of -5.35 kcal/mol and two hydrogen bonds. While this binding energy fell below the -5 kcal/mol threshold, indicating a spontaneous interaction, it represents a relatively low affinity when contextualized against the predicted therapeutic agents AMOPYROQUINE (-6.65 kcal/mol) and Genistein (-6.78 kcal/mol). These preliminary computational findings suggest that the environmental endocrine disruptor BPA has the potential to interact with HPSE, though its capacity to independently drive pathogenic effects in psoriasis requires careful experimental validation. Show in **Figures 8D, E** and **Table 3**.

**Table 3:** Details of molecular docking.

Gene name	Docking material	Hydrogen number	Binding energy(kcal/mol)
HPSE	AMOPYROQUINE	1	-6.65
HPSE	NARINGIN	3	-1.64
HPSE	Genistein	5	-6.78
HPSE	BPA	2	-5.35

### RT-qPCR validation of hub genes

To determine whether the psoriasis cell model had been successfully established, RT-qPCR was employed to assess the transcriptional levels of seven cytokine genes (differentiation markers) – IL-1β, IL-6, IL-8, S100A8/9, KRT6/10 – in M5-stimulated HaCaT cells. Results demonstrated that, compared to negative control cells, M5-induced HaCaT cells exhibited significantly elevated mRNA levels of IL-1β, IL-6, IL-8, S100A8/9, and KRT6; KRT10 was markedly reduced, indicating differentiation of HaCaT cells into hyperproliferative psoriatic keratinocytes under M5 stimulation. We further examined the transcriptional levels of five predicted hub genes using RT-qPCR. Results demonstrated that HPSE, PCLAF, and SPRR2C were significantly upregulated in M5-induced HaCaT cells. These findings were consistent with the hub gene predictions. Show in **Figure 9**.

**Figure 9:**
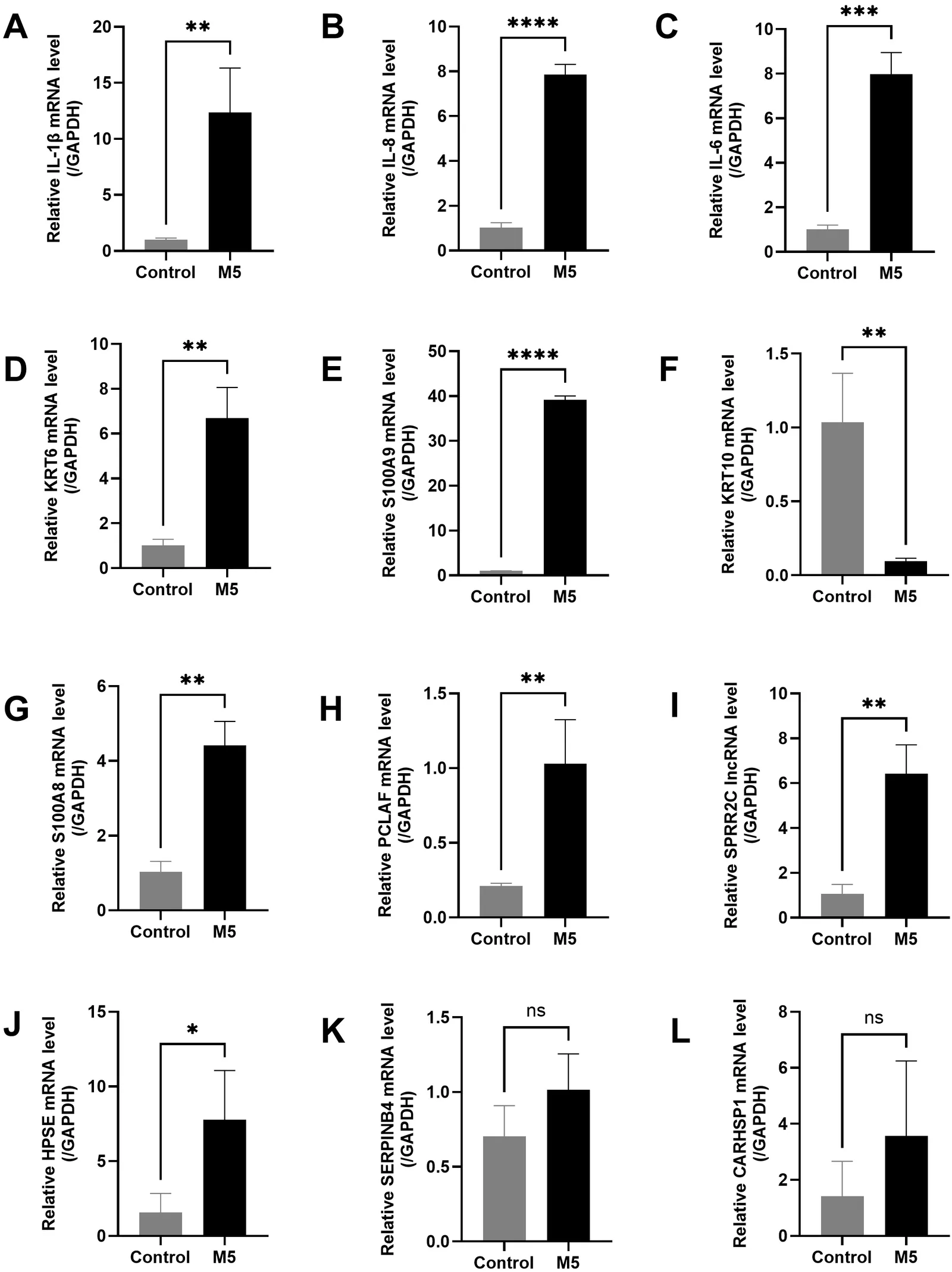
RT-qPCR results of hub genes and cytokine genes. **(A–L)** Relative mRNA expression levels of IL-1β **(A)**, IL-8 **(B)**, IL-6 **(C)**, KRT6 **(D)**, S100A9 **(E)**, KRT10 **(F)**, S100A8 **(G)**, PCLAF **(H)**, SPRR2C **(I)**, HPSE **(I)**, SERPINB4 **(K)**, and CARHSP1 **(L)** in Control and M5 groups. GAPDH was used as the internal control. Data are presented as mean ± SD (n = 3). *P < 0.05, **P < 0.01, ***P < 0.001, ****P < 0.0001; ns, not significant.

## Discussion

This study systematically screened and validated key pathogenic genes in psoriasis through the integration of bioinformatics, machine learning, and experimental validation. A total of 70 upregulated and 7 downregulated differentially expressed genes were identified across four training datasets. Further screening using LASSO and SVM-RFE machine learning methods identified five core hub genes (HPSE, PCLAF, SPRR2C, CARHSP1 and SERPINB4). These genes exhibited significantly elevated expression in psoriatic lesional skin compared with paired non-lesional skin in GSE30999 and demonstrated high discriminative value for lesional skin (AUC > 0.9). However, this does not imply diagnostic utility for distinguishing psoriasis from healthy skin or other inflammatory skin diseases. Immune cell infiltration analysis revealed their pivotal roles in regulating diverse immune cells including T cells, macrophages, and dendritic cells. Furthermore, the study constructed an mRNA-miRNA-lncRNA regulatory network elucidating how these hub genes are modulated. Molecular docking also predicted a potential interaction between the environmental pollutant bisphenol A (BPA) and HPSE, suggesting that BPA might influence psoriasis pathogenesis through HPSE; however, this remains a computational hypothesis that has not been experimentally validated. Drug prediction and molecular docking indicated HPSE exhibits the strongest binding affinity with Genistein. Finally, *in vitro* experiments confirmed significant upregulation of HPSE, PCLAF, and SPRR2C in M5-induced psoriasis cell models, providing preliminary transcriptional support for their involvement in psoriasis-related keratinocyte activation. KEGG enrichment analysis revealed significant enrichment of pathways including hepatitis C, Epstein-Barr virus (EBV) infection, and measles. While these are annotated as viral infections, their consistent enrichment in psoriasis transcriptomes is now widely recognized to reflect the overrepresentation of shared immune effector programs—notably, the type I interferon (IFN) response and antiviral restriction factors (15, 16). Multiple independent bioinformatics studies have demonstrated that ‘defense response to virus’ is among the most significantly enriched Gene Ontology terms in psoriatic lesions (17). This is explained by the fact that key antiviral genes (e.g., IRF7, IFIT1, IFI44L, MX1, ISG15) are not merely markers of infection but are intrinsic regulators of psoriatic inflammation, mediating keratinocyte-intrinsic immunity and crosstalk with Th17 responses (18). For instance, the hepatitis C pathway’s enrichment can be attributed to its overlap with IL-6, TNF, and STAT3 signaling networks central to psoriasis, as 389 shared genes have been identified between the two conditions (19). Thus, the presence of these viral pathways is not indicative of a broad, non-specific viral etiology. Instead, it highlights the molecular convergence of antiviral defense and autoimmune inflammation, where the same gene modules are co-opted in different pathological contexts.

Heparanase (HPSE)-a major functional enzyme expressed in mammalian cells-participates in the degradation of the extracellular matrix and the release of effector molecules bound to heparin sulphate (HS) by hydrolyzing HS (20–25). Has its 17β-estrogen-regulated expression in pathological conditions such as cancer and chronic inflammatory diseases (26). HPSE activity is frequently associated with extracellular matrix (ECM) remodeling, immune cell activation, and the release of chemokines anchored within the ECM network and on cell surfaces (27). Localized expression of HPSE synthase (upregulated by epithelial and vascular endothelial cells) at sites of inflammation has been validated in delayed-type hypersensitivity (28), vascular injury (29), chronic colitis (30), sepsis-associated mouse models of primary lung injury (31), and various human autoimmune and autoinflammatory diseases including rheumatoid arthritis (32), atherosclerosis (33, 34), inflammatory bowel disease (30, 35, 36), and psoriasis (37). Lerner I et al. results reveal that involvement of HPSE in the pathogenesis of psoriasis and suggest a role for the enzyme in facilitating abnormal interactions between immune (i.e., macrophages) and epithelial (keratinocytes) cellular subsets of the affected skin (37). Additionally, HPSE is the only gene whose target site can form a stable complex with BPA and overlaps with it. As an endocrine disruptor, BPA is used in the manufacture of polycarbonate plastics and the epoxy resins found in most canned foods and beverages (38). Such environmental exposure contributes to the onset of various autoimmune diseases, including psoriasis (39). BPA is independently known to activate inflammasomes and stimulate interferon signaling pathways in myeloid cells (40). These parallel inflammatory cascades could indirectly synergize with or exacerbate the HPSE/IL-17 axis (41) without strictly relying on the estrogen-regulated transcription of HPSE. Furthermore, BPA possesses weak estrogenic properties, which may explain its role in psoriasis via HPSE (42). See **Figure 10** for the mechanism diagram. While we propose a mechanistic chain where BPA’s weak estrogenic properties drive 17β-estrogen-regulated HPSE expression, ultimately feeding into an IL-17 positive feedback loop, we acknowledge this unified model is currently speculative. It is highly plausible that parallel or alternative pathways are involved. For instance, our molecular docking results suggest BPA may exert effects through direct, spontaneous binding to the HPSE protein itself, which could alter its enzymatic activity independently of transcriptional regulation via estrogen receptors.

**Figure 10:**
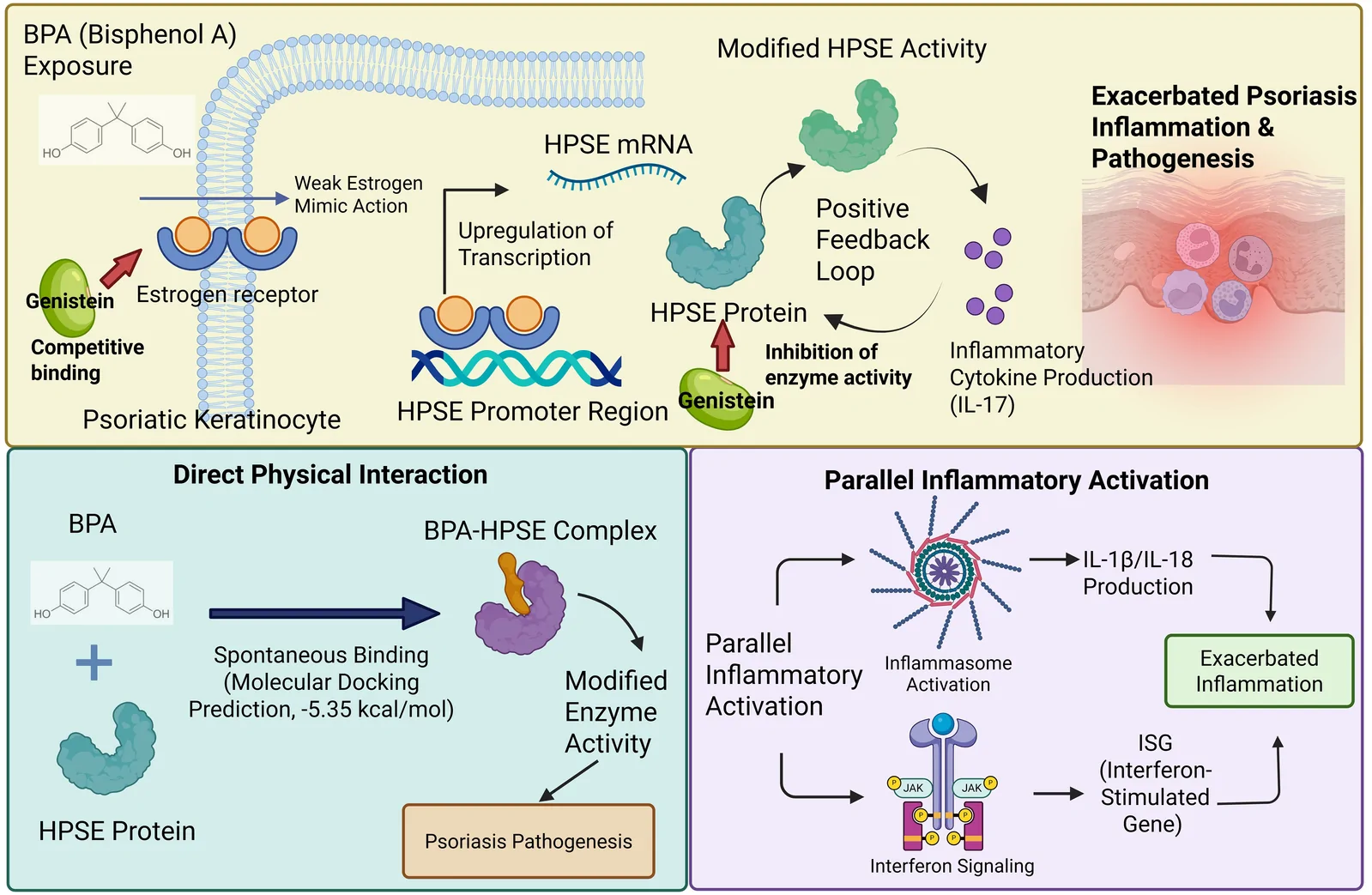
Schematic diagram of the BPA-HPSE-PSO mechanism.

We validated the specific antiestrogen agent Genistein as the drug with the strongest binding affinity to HPSE through molecular docking. Genistein is a well-characterized naturally occurring isoflavone (phytoestrogen) that acts as a selective estrogen receptor modulator (SERM) (43). Our docking results suggest it may directly bind to the HPSE protein, thereby potentially inhibiting its enzymatic activity and disrupting HPSE’s pathogenic role in psoriasis. Alternatively, it is well-established that Genistein can exert anti-estrogenic effects by competitively antagonizing the estrogen receptor (ER) (44)against more potent endogenous estrogens, which may indirectly downregulate HPSE expression (26). The clinical feasibility of this therapeutic avenue is highly promising. Interestingly, existing literature, including our referenced studies (38), frequently investigates the counteracting epigenetic and molecular effects of Genistein against environmental endocrine disruptors like BPA.

Furthermore, Genistein has been extensively documented for its potent anti-psoriatic and anti-inflammatory properties. *In vivo* and *in vitro* studies have demonstrated that Genistein significantly improves imiquimod (IMQ)-induced psoriasis-like skin lesions, inhibits the hyperproliferation of keratinocytes (HaCaT cells), and suppresses the expression of critical pro-inflammatory cytokines, including IL-17, IL-6, IL-23, and TNF-α, primarily by modulating the STAT3 and NF-κB signaling pathways (45, 46). Recent clinical and translational investigations have also reported reductions in clinical and biochemical scores in patients with mild to moderate psoriasis following Genistein administration, alongside a reduction in IL-17A-induced pathways in human epidermal keratinocytes (47). These collective findings robustly corroborate our computational prediction, underscoring the promising therapeutic potential of Genistein and HPSE-targeted interventions in the clinical management of psoriasis. Furthermore, the existing literature strongly supports the clinical feasibility of other representative drugs in this therapeutic class. Historically, multiple case reports have documented the unexpected remission of psoriasis in patients receiving the antiestrogen compound tamoxifen for breast cancer (48). Building on this, a recent open-label study demonstrated that 0.1 mg/ml topical tamoxifen gel significantly improved plaque psoriasis in patients who had previously failed mainstay topical and systemic therapies (49). Mechanistically, recent *in vivo* studies have shown that 17-β-estradiol promotes mannan-induced psoriasis-like skin inflammation via estrogen receptor-β (ER-β); importantly, the administration of an ER-β antagonist successfully decreased dermal γδ T cells and ameliorated the inflammatory response (50). To circumvent potential systemic side effects of oral antiestrogens, researchers are currently advancing liposomal topical formulations to enhance epidermal delivery. These collective clinical and experimental findings robustly corroborate our prediction, underscoring the promising therapeutic potential of antiestrogens and HPSE-targeted interventions in the clinical management of psoriasis. HPSE exhibits significant functional roles across multiple analytical levels, suggesting it may serve as a pivotal molecule linking environmental exposure, immune regulation, and psoriasis pathogenesis.

The PCLAF (PCNA Clamp Associated Factor; also known as KIAA0101 or PAF) was identified as a proliferating cell nuclear antigen (PCNA)-interacting protein modulating DNA repair and replication (51). In our study, PCLAF was significantly upregulated in M5-induced HaCaT cells and showed strong positive correlations with nearly all T cell subsets and myeloid immune cells. PCLAF, as a PCNA-binding protein, primarily participates in DNA replication, cell cycle regulation, and cell proliferation (52), which aligns closely with the pathological feature of excessive proliferation in psoriatic keratinocytes (53). Its widespread immunopositivity suggests PCLAF may indirectly promote psoriatic inflammation by regulating immune cell infiltration. Within the competing endogenous RNAs(ceRNA) network, PCLAF interacts with 42 miRNAs and 216 lncRNAs. Although smaller in scale than HPSE, this network still demonstrates considerable regulatory complexity.

Small proline rich protein 2C (pseudogene), denoted as SPRR2C, encodes a lncRNA rather than a mRNA as its transcript (54). This study revealed through bioinformatics analysis and experimental validation that SPRR2C is significantly upregulated in psoriasis cell models. Previous research has also confirmed that SPRR2C modulates IL-22-stimulated HaCaT cell phenotypes via the miR-330/STAT1/S100A7 axis (55). SPRR2C is a key molecule in keratinocyte differentiation and skin barrier formation, with its increased expression directly linked to psoriatic barrier dysfunction and abnormal keratinization processes (56). Notably, our competing endogenous RNA(ceRNA) network analysis did not identify corresponding upstream miRNAs for SPRR2C. This absence suggests that its robust upregulation in psoriatic lesions may be governed by alternative regulatory mechanisms, particularly epigenetic modifications. The Epidermal Differentiation Complex (EDC), where the SPRR gene cluster resides, is highly susceptible to epigenetic remodeling (57). Previous pivotal studies have demonstrated that extensive DNA hypomethylation and activating histone modifications within the EDC region actively drive the coordinated overexpression of these genes during keratinocyte hyperproliferation and chronic inflammation (58). Furthermore, its transcription may be directly stimulated by key inflammatory transcription factors activated by the psoriatic cytokine storm. As the sole lncRNA marker among the five genes, lncRNA exhibits higher tissue specificity and may emerge as a more precise diagnostic target than mRNA in future applications (59).

Calcium-regulated heat shock protein 1 (CARHSP1) has been identified as a member of the cold shock domain (CSD) protein family, participating in the regulation of ribosomal translation, mRNA degradation, and transcription termination rates (60). Squamous cell carcinoma antigens 1 and 2 (SCCA1 and 2, SERPIN B3 and B4) are members of the ovalbumin serpin (ov-serpin)/clade B serpin family (61) and were originally discovered as tumor-specific antigens in the uterine cervix (62). Both CARHSP1 and SERPINB4 are core psoriasis hub genes, significantly upregulated in the validation dataset (p<0.001) with AUC values of 0.906 and 0.986 respectively, demonstrating strong diagnostic specificity. However, no significant expression differences were observed in M5-induced HaCaT cell models. We postulate that this discrepancy is driven by two distinct mechanisms reflecting the well-documented limitations of *in vitro* monocultures. First, regarding CARHSP1, single-cell transcriptomic data from the Human Protein Atlas (HPA) and previous functional studies confirm that it is abundantly expressed in immune cells, particularly macrophages and T cells (63). Given the massive immune infiltration in psoriatic lesions, the significant upregulation of CARHSP1 in clinical bulk datasets is predominantly driven by the influx of this immune compartment, which is absent in isolated HaCaT cultures. Second, regarding SERPINB4, although it is an epithelial-specific factor, its explosive upregulation *in vivo* relies on prolonged exposure to a highly complex inflammatory network. As previously demonstrated by Gudjonsson et al., *in vitro* cytokine-stimulated keratinocyte models only partially overlap with the *in vivo* psoriatic transcriptome due to the lack of dynamic stromal-immune crosstalk (64). The simplified 12-hour M5 stimulation in a HaCaT monoculture lacks the full-scale synergistic cytokine milieu (65) required for its complete induction. Mechanistically, the RNA-binding protein CARHSP1 identified and selectively bound to the mRNA of IL-17 receptor A (IL-17RA), resulting in the increased expression of both IL-17RA mRNA and protein. Downregulating expression of CARHSP1 shortened the half-life of IL-17RA mRNA and reduced its expression (60). While SERPINB4 participates in psoriasis as a downstream molecule of IL-22 and IL-17 (64, 66, 67). Beyond the individual pathways of apoptosis, pyroptosis, and necroptosis, psoriasis involves a complex interplay among these regulated cell death modalities, collectively termed PANoptosis. Emerging evidence indicates that PANoptosis signaling can amplify immune responses in psoriatic skin by promoting the release of pro-inflammatory cytokines and danger signals from keratinocytes and immune cells (68). Given our observation that the hub genes are associated with multiple immune cell populations and inflammatory pathways, it is plausible that some of these genes participate in or respond to PANoptotic processes. For example, HPSE-mediated extracellular matrix remodeling may influence the local microenvironment in which PANoptosis occurs, while CARHSP1, an RNA-binding protein, could affect the stability of transcripts involved in cell death signaling. Further studies are needed to determine whether the five hub genes intersect with PANoptosis pathways and whether targeting HPSE or related genes could modulate this form of inflammatory cell death.

This study retains several limitations. Firstly, data analysis relies entirely on transcriptomic microarray data from public databases. Future integration of multi-omics data, including single-cell sequencing and epigenomics, will enable more precise elucidation of the cell type-specific functions of pivotal genes. In this context, a specific limitation regarding our immune infiltration analysis involves the application of CIBERSORT for the deconvolution of bulk tissue microarrays. While robust, this algorithm possesses inherent limitations that must be contextualized. First, its accuracy is sensitive to the LM22 reference signature matrix, which is derived from purified cell populations and may not entirely capture the altered transcriptomic profiles of immune cells residing within the highly inflamed psoriatic skin microenvironment. Second, potential collinearity among closely related immune cell signatures can complicate the precise mathematical distinction between overlapping cell lineages. Finally, CIBERSORT is fundamentally limited to the 22 broad immune subsets defined in its reference panel; consequently, it lacks the resolution to detect intermediate, transient, or highly specialized disease-specific immune states—such as specific pathogenic Th17 subsets—that are not represented in the matrix. Furthermore, the associations identified between hub gene expression and specific immune cell populations are strictly correlative and do not establish causation. We acknowledge that these correlations are susceptible to confounding variables, including inter-sample differences in clinical disease severity, variations in bulk tissue composition (such as the relative proportion of structural tissue stroma), and the influence of shared upstream inflammatory regulators that may independently but simultaneously drive both gene expression and immune cell recruitment. Secondly, although the expression of pivotal genes was screened via machine learning and experimentally validated, their specific molecular mechanisms and upstream/downstream signaling pathways in psoriasis remain unclear. This necessitates further exploration through functional experiments such as gene editing and overexpression. Moreover, while the psoriasis cell model employed (M5-induced HaCaT cells) mimics certain disease characteristics, it falls short of replicating the complex *in vivo* microenvironment. Future utilization of organoids or psoriasis animal models will provide more human-relevant functional validation platforms. Furthermore, due to the retrospective nature of the public transcriptomic databases utilized, detailed clinical metadata such as Psoriasis Area and Severity Index (PASI) scores were unavailable for individual samples. Fourth, our experimental validation was conducted exclusively at the mRNA level by RT-qPCR. Although transcriptional upregulation is a critical indicator, it does not account for post-transcriptional regulatory mechanisms, variations in translational efficiency, or protein degradation rates, and therefore does not directly confirm a corresponding increase in functional protein abundance or enzymatic activity. Notably, for HPSE, elevated protein expression and heparanase activity in psoriatic skin have been previously demonstrated by Lerner et al. using immunohistochemistry and enzymatic assays, which provides orthogonal support for our mRNA-level finding. For the remaining hub genes—PCLAF, SPRR2C, CARHSP1, and SERPINB4—protein-level evidence in the context of psoriasis remains largely absent. Future studies employing Western blot, immunohistochemistry, ELISA, or targeted proteomics on clinical psoriatic specimens and relevant cell models are essential to confirm the translational relevance of our mRNA findings and to establish these genes as functional pathogenic drivers rather than mere transcriptional markers. A further limitation concerns the heterogeneity of the control tissues across the training datasets. GSE13355 and GSE166388 used healthy normal skin (NN), whereas GSE34248 and GSE41662 used paired non-lesional (NL) skin. NL skin is a pre-psoriatic, sensitized microenvironment and is not biologically equivalent to NN skin. Our strict intersection approach was designed to identify robust, universally present lesional effector genes, but it consequently acts as a downstream effector filter, favoring terminal inflammatory and hyperproliferative signals while potentially missing early initiating factors that are only detectable against a true healthy baseline. Accordingly, we explicitly interpret the five hub genes as lesion-associated markers reflecting overt lesional activity, rather than as disease-initiating genes or as diagnostic markers for distinguishing psoriasis from healthy skin. A relaxed filtering strategy confirmed that these five genes remain consistent across baselines, supporting their reliability as common downstream effectors. Future studies using only NN controls are needed to capture the earliest molecular events in psoriasis pathogenesis. We state that the reported AUC values reflect the genes’ capacity to distinguish active, severe lesional skin from paired non-lesional skin in patients with established moderate-to-severe psoriasis. Their performance in a more clinically realistic setting—such as differentiating psoriasis from healthy skin, mild psoriasis from other inflammatory dermatoses, or detecting early-stage disease—remains untested and is likely lower. Consequently, a direct correlation between the expression levels of these hub genes and clinical disease severity could not be established in the current study. Future prospective cohorts with comprehensive clinical documentation are required to further validate the clinical relevance of these targets. Finally, the proposed interaction between the environmental pollutant BPA and HPSE relies entirely on in silico molecular docking predictions. No *in vitro* BPA-exposure experiments were performed in this study to demonstrate whether BPA can directly alter HPSE expression levels or enzymatic activity in keratinocytes. Molecular docking provides a static, rigid-body approximation that does not account for protein flexibility, solvent effects, or the dynamic cellular context; therefore, the predicted binding—even if thermodynamically plausible—may not occur in a biologically meaningful way. Consequently, the environmental pathogenic axis postulated in **Figure 10** remains a computational hypothesis and should not be interpreted as an established mechanism. Rigorous biophysical techniques (e.g., surface plasmon resonance, isothermal titration calorimetry) and functional assays in BPA-exposed keratinocyte models are essential future steps to confirm or refute this proposed link. A further limitation concerns the strategy used for cross-cohort integration. Candidate genes were identified by taking the strict intersection of DEGs across the four training datasets. This approach is conservative and is not equivalent to batch-effect correction or formal meta-analysis. In particular, GSE166388 contains only four disease samples and four control samples and may strongly restrict the final set of 77 intersecting genes. Leave-one-dataset-out sensitivity analysis and effect-size-based meta-analysis would strengthen the robustness assessment, but these analyses were not performed in the current study. Future studies should apply formal meta-analytic methods and larger harmonized cohorts to further validate the identified hub genes. The mRNA-miRNA-lncRNA network was constructed solely from target prediction databases and was not filtered or validated using miRNA or lncRNA expression data from psoriatic tissues. The expected negative correlations between miRNAs and target mRNAs and positive correlations between lncRNAs and mRNAs were not examined. Consequently, the network represents predicted interactions only and does not establish a functional ceRNA mechanism. Future studies should integrate matched expression data to refine and validate these predicted interactions. Finally, a notable limitation of this study is that the interaction between BPA and HPSE relies on computational molecular docking predictions. Although the docking results suggest the presence of spontaneous interactions, the rigid-body docking method employed provides only static snapshots and fails to account for protein flexibility, solvent interactions, or dynamic conformational changes *in vivo*; furthermore, this study currently lacks functional experimental validation. The direct effects of BPA exposure on HPSE expression, enzymatic activity, and subsequent downstream signaling in keratinocytes remain to be elucidated. Future studies employing targeted *in vitro* cellular assays (such as keratinocyte BPA exposure models) and rigorous biophysical techniques (including surface plasmon resonance [SPR] or isothermal titration calorimetry [ITC]) are strictly warranted to experimentally validate this computationally predicted pathogenic link. Looking ahead, the five hub genes identified in this study offer valuable insights for mechanistic research and therapeutic development in psoriasis. Subsequent work will focus on: elucidating the specific functions of each gene in more advanced models; and advancing the translation of HPSE-targeted drugs (such as anti-estrogen drugs) from computational prediction to preclinical research. These endeavors hold promise for delivering novel precision diagnostic and therapeutic strategies for psoriasis patients.

## Conclusion

This study systematically identified five core psoriasis hub genes—CARHSP1, HPSE, PCLAF, SERPINB4, and SPRR2C—through the integration of bioinformatics, machine learning, and experimental validation. These genes show strong discriminatory value for established psoriatic lesions and are associated with immune infiltration, cell-cycle activity, and skin barrier alterations. However, the present data support their role as lesion-associated markers rather than as proven drivers of disease initiation. The study further constructed an mRNA-miRNA-lncRNA regulatory network, predicting via computational docking that the environmental pollutant BPA may bind to HPSE. This suggests a potential environmental pathogenic mechanism that requires strict experimental validation in future studies. Concurrently, computational docking predicted the potential of anti-oestrogen drugs as HPSE-targeted candidates, although experimental validation is lacking. This establishes a comprehensive pathogenic framework linking genetic susceptibility, immune dysregulation, environmental exposure, and targeted treatment, providing a framework for future investigation of lesion-associated biomarkers and targeted therapeutic strategies in psoriasis, while acknowledging that the current evidence supports an associative rather than causal role for the identified genes.

## Data Availability

The datasets presented in this study can be found in online repositories. The names of the repository/repositories and accession number(s) can be found in the article/**Supplementary Material**.
